# Whey as an Alternative Nutrient Medium for Growth of *Sporosarcina pasteurii* and Its Effect on CaCO_3_ Polymorphism and Fly Ash Bioconsolidation

**DOI:** 10.3390/ma14102470

**Published:** 2021-05-11

**Authors:** Sandra Chaparro, Hugo A. Rojas, Gerardo Caicedo, Gustavo Romanelli, Antonio Pineda, Rafael Luque, José J. Martínez

**Affiliations:** 1 School of Chemical Sciences, Faculty of Sciences, Pedagogical and Technological University of Colombia, 150001 Tunja, Colombia; patricia.chaparro@uptc.edu.co (S.C.); hugo.rojas@uptc.edu.co (H.A.R.); gerardo.caicedo@uptc.edu.co (G.C.); 2 Research and Development Centre on Applied Sciences “Dr. Jorge Ronco” (CCT-La Plata-CONICET, CIC-PBA), National University of La Plata, 1900 La Plata, Argentina; gpr@quimica.unlp.edu.ar; 3 Departamento de Química Orgánica, Universidad de Córdoba, Ctra NNal IV-A, Km 396, E-14014 Córdoba, Spain; q82pipia@uco.es; 4 Scientific Center for Molecular Design and Synthesis of Innovative Compounds for the Medical Industry, Peoples Friendship University of Russia (RUDN University), 117198 Moscow, Russia

**Keywords:** biogenic CaCO_3_, polymorphism, ureolytic bacteria, whey

## Abstract

Whey in large quantities can cause environmental problems when discarded, because it reduces dissolved oxygen and aquatic life. Nonetheless, it could be used as an easily available and economical alternative to reduce culture medium costs in microbially induced calcium carbonate precipitation (MICP). In this work, a native *Sporosarcina pasteurii* was isolated and then cultured by using different proportions of whey (W) in nutrient broth (NB). The solids were characterized by XRD, FT-IR, TGA, and SEM. The potential applications in bioconsolidation were also studied. Whey concentration was directly related to CaCO_3_ production. Higher whey concentrations reduced calcium carbonate purity to nearly 80%. All experiments showed calcite and vaterite fractions, where a whey increment in the media increased calcite content and decreased vaterite content, causing a decrease in crystal size. MICP improved compressive strength (CS) in sand and fly ash. The best CS results were obtained by fly ash treated with 25 W-75 NB (37.2 kPa) and sand with 75 W-25 NB (32.1 kPa). Whey changed crystal polymorphism in biogenic CaCO_3_ production. Material bioconsolidation depends on the CaCO_3_ polymorph, thus fly ash was effectively bioconsolidated by crystallization of vaterite and sand by crystallization of calcite.

## 1. Introduction

Calcium carbonate precipitation (MICP) indicates the mineral formation from an oversaturated solution, mediated by the metabolism of microorganisms [1]. In MICP, cells secrete metabolic products (CO_3_^2−^) that react with the ions (Ca^2+^) present in the environment with subsequent mineral precipitation. Such substances act as cementing materials and are commonly known as “biocement” [2]. The pathways reported to calcium carbonate formation include photosynthesis [3], urea hydrolysis [4,5,6], anaerobic sulfide oxidation [7], and by extracellular polymeric substances [3,8].

Urea hydrolysis is the most extensive method of biogenic CaCO_3_ production because urease is present in some microorganisms [9,10,11]. One of the objectives of MICP technology is to isolate and select bacterial strains with increased urease activity, higher stability, and greater tolerance to high ammonium concentrations [12]. There are many ureolytic microorganisms, such as the genus *Bacillus*, that have a higher urease content [13].

*Sporosarcina pasteurii* is a Gram-positive bacterium used in MICP because of its high enzyme content (which reaches almost 1% by dry weight), and its zero pathogenicity [9,14,15]. This bacterium grows in different kinds of soils and can resist extreme conditions of pH and temperature. For *S. pasteurii* growth, some authors used nutrient broth [4,16,17], yeast extract [18,19,20], heart brain infusion broth [21], peptone [22], or meat extract [22], all of which were supplemented with urea. However, cheaper substitutes should be sought, due to the necessity of large-scale production. Consequently, it is necessary to implement the use of available, inexpensive agro-industrial waste to reduce culture medium costs [23].

Some alternatives to replacing nutrients and achieving a level of urease activity are torula yeast (*Cyberlindnera jadinii*), brewery waste yeast, Vegemite^®^, and acetate (which lowered production costs by 95%) [19]. Additionally, media such as fermented maize liquor [24] and lentils [12] have been used.

Whey is the watery part of milk that results from cheese coagulation in industrial processes. It has a significant carbohydrate, protein and mineral content [25]. When a large amount of whey is discarded as effluent it causes an environmental problem [26] and reduces dissolved oxygen and aquatic life as consequence [27].

There are few reports in the literature on MICP that include the use of whey. Achal et al. [28] studied microbial concrete production using lactose mother liquor (LML) as a different nutrient for bacteria. They added only 10% of this by-product to a yeast extract and nutrient broth medium. The compressive strength of the cement mortar was increased by *S. pasteurii* in all the media used compared to the control (sand with cement 3:1 % *w*/*w*). No significant difference in bacterial growth, urease production, and mortar compressive strength with LML was found, suggesting that this is another source of typical media. Cuzman et al. [29] tried different nutrients for bacteria such as lactose mother liquor, buttermilk, yeasts, liquid and dried yeasts from breweries, liquid bakery yeast, and Vegemite^®^. They reported that whey and buttermilk waste were potential nutrients for biotechnological applications with *S. pasteurii*. They did not study CaCO_3_ crystals or their possible applications. Grabiec et al. [30] studied CaCO_3_ biodeposition with *S. pasteurii* in the modification of recycled concrete aggregate, and whey was used as an alternative culture medium. They showed that CaCO_3_ biodeposition led to the reduction in aggregate water absorption.

These previous works did not study whey incorporation in different quantities, calcium carbonate production or the type of crystal produced. This paper shows the results of using whey as an inexpensive nutrient source for ureolytic bacteria and its effect on the polymorphism of CaCO_3_ precipitate. Additionally, compressive strength changes were analyzed using different material types and whey addition percentages.

## 2. Materials and Methods

### 2.1. Bacterial Isolation

Bacteria were isolated from agricultural soils of Sotaquirá and Nobsa (Boyacá, Colombia), following a previously established protocol [31]. Urease activity was determined by the conductivity method [19]. The bacterium with higher urease activity was molecularly identified by amplification of the 16S ribosomal gene [32].

### 2.2. Culture Media

To increase urease activity and calcium carbonate production, experiments with three types of media were performed: (1) yeast extract (10 g/L), ammonium sulfate (AS, 20 g/L), sodium acetate (SA, 8.2 g/L), and urea (U, 60 g/L); (2) tryptone (10 g/L), AS (20 g/L), SA (8.2 g/L), and U (60 g/L); and (3) nutrient broth (NB, 13 g/L) and U (30 g/L) [33]. Tests were carried out for 24 h at 30 °C and 200 rpm. Then, CaCl_2_ solution (30 g/L) was used to precipitate calcium carbonate at pH 7.2. Urease activity, biomass and CaCO_3_ production were recorded. Experiments were done by triplicate. Urease activity was determined by the conductivity method [19]. Biomass was measured at 600 nm using a spectrophotometer Bio Whittaker ELX 80834 (BioTek Instruments Inc., Winooski, VT, USA). Calcium carbonate production was measured by weight difference.

### 2.3. Effect of Whey on CaCO_3_ Crystals and Bioconsolidation Treatment

The influence of different whey concentrations on nutrient broth during 24 h at 30 °C and 200 rpm was assessed by the fermentation of bacterial cultures for 24 h. Table S1 indicates whey composition and its comparison with other studies. The media were labelled as 100 NB, 25 W-75 NB, 50 W-50 NB, 75 W-25 NB, 100 W, where the number indicates the percentage of whey (W) or nutrient broth (NB) used. Then, calcium chloride (30 g/L) was added and mixed for 3 h The initial and final pH values were 6.5 ± 0.5 and 8.5 ± 0.3, respectively. The resulting precipitate was collected and centrifuged (10 min at 6000 rpm) and the solid was recuperated and washed three times with distilled water. Next, 20 mL of sodium hypochlorite (10%) was added and allowed to stand for 6 h. Afterward, four water and ethanol washes were done. Finally, the solid was dried at 70 °C for 24 h.

### 2.4. Characterization of Biogenic CaCO_3_ Obtained Using Whey at Different Proportions in Nutrient Broth

The precipitates obtained were evaluated by X-ray diffraction (XRD, Malvern, Westborough, WA, USA,) with Co Kα radiation (k = 1.789010 Å) and scanning from 20 to 70° 2θ (PANalytical X’Pro instrument, Malvern, Westborough, WA, USA). XRD peaks were identified using JCPDS data file. The minerals present in the precipitate were quantified using a Rietveld refinement in the X’Pert HighScore Plus© software with the PDF-4-2012 database and the American Mineralogist Crystal Structure Database [34].

After whey treatments, the Brunauer–Emmett–Teller (BET)-specific surface area, pore volume and size were measured using a physisorption method (ASAP 2020, Micromeritics Instrument Corporation, Norcross, GA, USA). CaCO_3_ obtained from different whey treatments was characterized by FT-IR spectra in Thermo Scientific Nicolet iS50 equipment in a range of 3000 to 500 cm^−1^. Thermogravimetric analysis (TGA) of CaCO_3_ obtained was performed using a Setaram Labsys EVO instrument at a heating rate of 10 °C/min in an air flow of 30 mL/min. To study the role of whey addition, calcium carbonate crystals were observed under a scanning electron microscope (SEM, Zeiss, Oberkochen, Germany). The samples were gold-coated with a sputter coating machine (Quorum Q150 R-ES, Zeiss, Oberkochen, Germany) and examined using Carl Zeiss EVO-MA10 equipment (Zeiss, Oberkochen, Germany).

### 2.5. Tests of Sand and Fly Ash Bioconsolidation

Sand and fly ash were used as materials for bioconsolidation. Material composition is presented in Table S2. Samples were autoclaved at 121 °C for 45 min. They were then placed inside columns and compacted. Plastic columns that were 3 cm in diameter and 10 cm in height were used. Bacterial culture, cementation solution (calcium chloride (30 g/L) and urea solution (30 g/L)) were interspersed using a surface percolation technique. Sand and thermoelectric ashes were treated with separate whey concentrations (two materials, five concentrations and triplicate: 30 samples). Sand and ash controls were packed in columns with sterile materials mixed with media only (without *S. pasteurii*). Treatment was performed by introducing 50 mL of *S. pasteurii* culture and cementation solution (50 mL) into columns every 12 h for 5 days. Afterward, all columns stayed at 20 °C for 28 days before they were taken out of their molds. Finally, all samples were analyzed using a compression testing machine [35]. TEM micrographs of bioconsolidated sand and fly ash were taken using a JEOL JEM 14000 High Resolution Transmission Electron Microscope (JEOL Peabody, MA, USA). Samples were suspended in ethanol and placed on a copper rack before analysis.

### 2.6. Statistical Analysis

All the experiments were carried out in triplicate. Analysis of variance (ANOVA) was performed, and Tukey’s test was applied when statistical significance was found.

## 3. Results and Discussion

### 3.1. Bacterial Isolation

**Figure 1:**
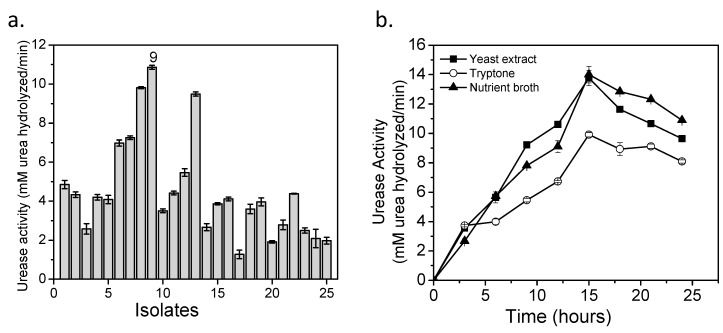
(**a**) Ureolytic bacteria isolated from different soils of Boyacá. (**b**) Effect of growth media on urease activity of *S. pasteurii.*

Twenty-five bacteria were isolated. Their urease activity (UA) varied between 1.3 and 10.8 mM of urea hydrolyzed/min (Figure 1a). Statistical analysis showed significant differences between evaluated isolates (F: 1342.3 ˃ F_crit_: 1.9; P = 2.41 × 10^−33^), and Tukey’s test indicated that isolate 9 had the highest urease activity. This isolate was identified as *Sporosarcina pasteurii* of Firmicutes phylum (99% similarity). *S. pasteurii* is a Gram-positive bacterium [13,36,37] and it hydrolyzed 29 mM urea/min [19].

### 3.2. Culture Media

Yeast extract and nutrient broth showed similar behavior in UA (Figure 1b). The best value was obtained in nutrient broth, 14.0 mM urea hydrolyzed/min at 15 h. These results are similar to *S. pasteurii* ATCC 11859 (13.7 mM urea hydrolyzed/min) [19] and higher than *S. pasteurii* WJ-2 (3.8 mM urea hydrolyzed/min) [38]. The CaCO_3_ yields were: 76%, 86%, and 99% for yeast extract, tryptone, and nutrient broth, respectively. There is a direct relation between UA and CaCO_3_ production [19], so it was expected that yeast extract would present the best mineral content. Nevertheless, the better results in nutrient broth confirmed that *S. pasteurii* adapted better to this medium to produce high urease amounts. UA and CaCO_3_ production showed statistical differences in ANOVA tests (UA F: 419.3 ˃ F_crit_: 5.2; CaCO_3_ F: 17.1 ˃ F_crit_: 5.2).

### 3.3. Effect of Whey in Nutrient Broth on Biogenic CaCO_3_

**Figure 2:**
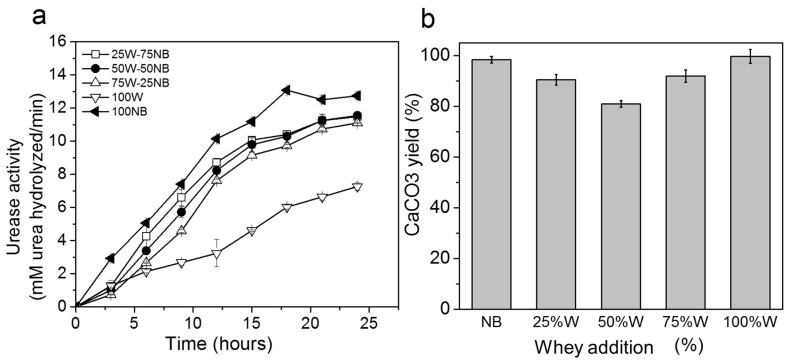
Effect of whey at different proportions in nutrient broth on (**a**) urease activity, and (**b**) CaCO_3_ yield.

The effect of whey in nutrient broth on urease activity and CaCO_3_ production is shown in Figure 2a,b, respectively. Significant statistical differences were found in the treatments (UA p_value_: 2.38 × 10^−11^; CaCO_3_ p_value_: 1.03 × 10^−15^). The use of pure whey decreased considerably with urease activity, in comparison to the assays containing nutrient broth, due to the high concentration of proteins in whey that are difficult to assimilate by microorganisms [27]. However, calcium carbonate production was similar in whey (100 W) and nutrient broth (100 NB) due to the excess of calcium ions. Although W-NB mixes presented similar urease activity, close to 100, CaCO_3_ production depended on protein proportion, where 50 W-50 NB had the lowest precipitate formation.

### 3.4. Characterization of Biogenic CaCO_3_ Obtained Using Whey at Different Proportions in Nutrient Broth

**Figure 3:**
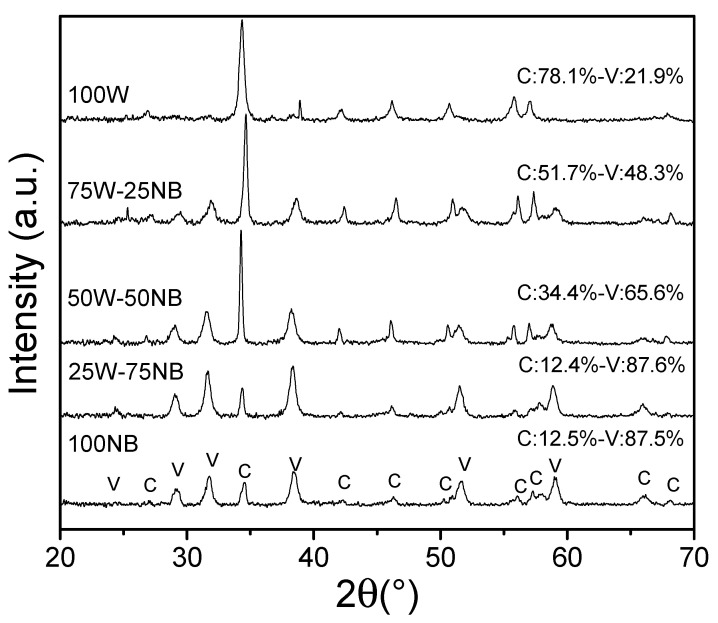
XRD of whey effect on biogenic CaCO_3_ production.

It has been reported that the crystal morphology of microbially induced calcium carbonate depends on the microorganism, calcium source, and growth media composition [6,39]. XRD results revealed calcite and vaterite fractions in all experiments (Figure 3).

From the Rietveld refinement of the XRD patterns, Figure 3 also presents the semi-quantitative ratios of the crystalline phases of both vaterite and calcite for all the precipitates. The weighted residual profile of the refinements (R_wp_) was below 10% and none presented any variation above 20% with respect to the expected values (R_exp_). These parameters determined that the refinement models were good to accept the values obtained [40]. In the precipitate obtained with only nutrient broth, *S. pasteurii* produced a high content of vaterite (87.5%). When whey concentration increased, calcite content increased up to 78.1% for 100 W.

According to different authors, vaterite grains agglomerate into larger particles that transform into calcite. In aqueous solution (culture), the reaction mechanism consists of three phases: (1) a progressive dissolution of small vaterite crystals on the grain’s surface, (2) generation of cubic calcite micro-germs (more stable than calcite), and (3) precipitation of cubic calcite providing rhombohedral forms [41]. Furthermore, it is recognized that the nucleation frequency and crystal size are both functions of supersaturation (first) and then the effect of impurities (second). When nucleation takes place in the bacterium cell surface, the supersaturation in basic medium begins with the exclusive formation of vaterite. A higher amount of impurities modifies the crystal growth morphology by interacting with the environment, which changes their size and structure type [42], providing evidence that whey promotes the production of calcite with the lowest crystallite sizes. This fact is probably due to the presence of protein residues in whey, and consequently, this higher impurities content causes preferential precipitation towards calcite. This also reduces the specific surface area (SSA) and increases others such as pore size and pore volume (Table 1). The presence of protein residues was verified with FT-IR and TGA results. Natural calcium carbonate has an SSA from 4 to 10 m^2^/g [43] and precipitated CaCO_3_ from 17 to 25 m^2^/g [44] and 27 m^2^/g [45], which is similar to biogenic CaCO_3_ obtained in the assay 100 NB.

Infrared transmission spectra of CaCO_3_ obtained with different whey additions show strong absorption bands, related to calcite and vaterite crystals. The assignation of these bands has been broadly discussed by Al et al. [46] and Andersen et al. [47], and some of these peaks can be seen in the spectra (Figure S1a, Table S3). The proportion of the band changes with the increase in whey in nutrient broth, confirming the reduction in vaterite content and the increase in calcite phase. The presence of protein residues can be evidenced by the amide bands in Figure S1b.

**Figure 4:**
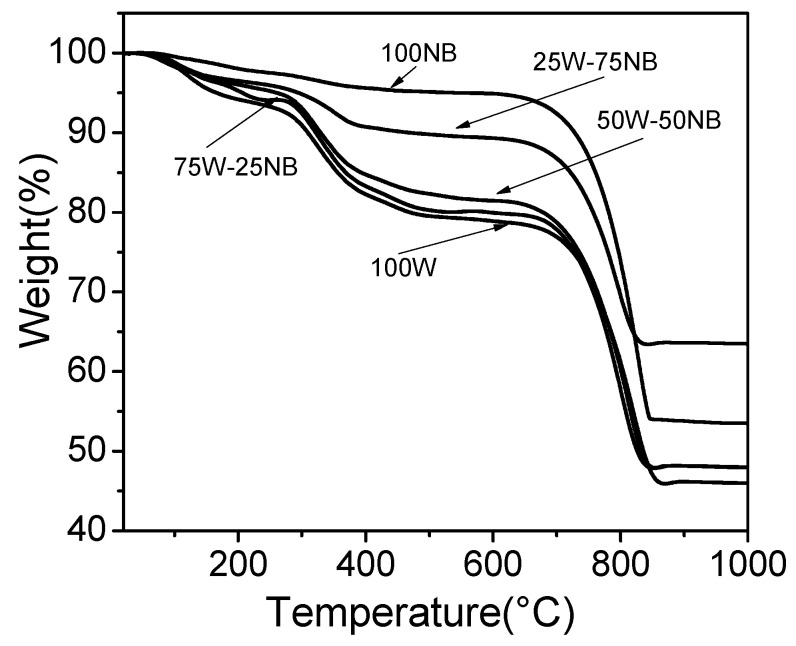
Thermograms of biogenic CaCO_3_ using different proportions of whey in nutrient broth.

The incorporation of whey in nutrient broth causes a decrease in CaCO_3_ purity, as can be seen in the results of thermogravimetric analysis (Table 1). TGA curves (Figure 4) reveal two water losses between 50 and 100 °C, and 100 and 250 °C. In the first step, bound water goes out, and in the second, structural water is lost. Weight loss in the range of 290 to 480 °C can be due to both loss of water and vaterite transformation to calcite that occurs at 488 °C [48]. Vaterite can transform into cubic calcite under heating conditions and in the absence of oxygen. In the literature, peaks of 481 and 488 °C are attributed to the transformation of vaterite into calcite [41]. This process implies the loss of weight shown in Figure 4. Treatments with high whey concentrations demonstrate weight loss due to the presence of calcite, which contrasts with treatment with 100 NB that has low calcite quantity. The elimination of organic matter can be revealed also in this range of temperature, explaining the highest weight loss. In conclusion, whey addition to nutrient broth decreases calcium carbonate purity, possibly due to the organic matter remaining in the solid, which could be verified with TGA and FT-IR.

**Figure 5:**
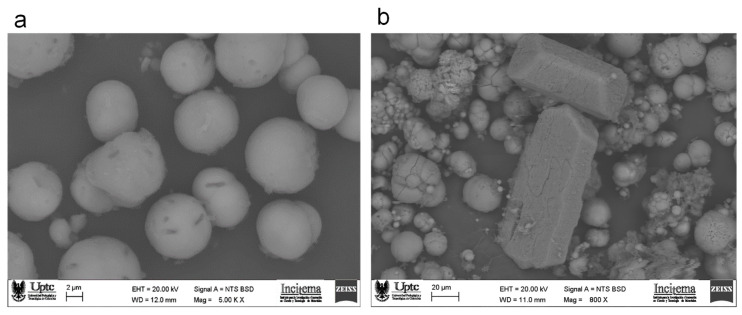
SEM micrographs of biogenic CaCO_3_ obtained with (**a**) nutrient broth (100 NB) shows vaterite crystals, and (**b**) whey (100 W) with calcite crystals.

Figure 5 shows SEM images of biogenic CaCO_3_ obtained with nutrient broth (100 NB) and whey (100 W). On the first micrograph (Figure 5a), crystals have a spherical shape, although this could be related to vaterite morphology [49], it is evident by XRD results that the presence of calcite is in minor proportion. This vaterite phase is more soluble and has a lower density compared to calcite, which is why it is not highly recommended for soil bioconsolidation and crack repair [6]. The elongated holes that can be seen on the crystal surface correspond to bacterial cells that act as nucleation sites for crystal growth, which indicates their biological origin [50]. Bacteria were in intimate contact with crystals, demonstrating that cells served as nucleation sites during the MICP process [51]. Figure 5b shows a typical SEM image of CaCO_3_ crystals obtained with 100% of whey that preferentially correspond to calcite with a rhombohedral structure [52]. In the present study, *S. pasteurii* and calcium chloride produced vaterite but whey incorporation in the nutrient broth increased calcite content. This confirms that strain, media and calcium type produced a defined crystal phase.

### 3.5. Bioconsolidation of Sand and Fly Ash

**Figure 6:**
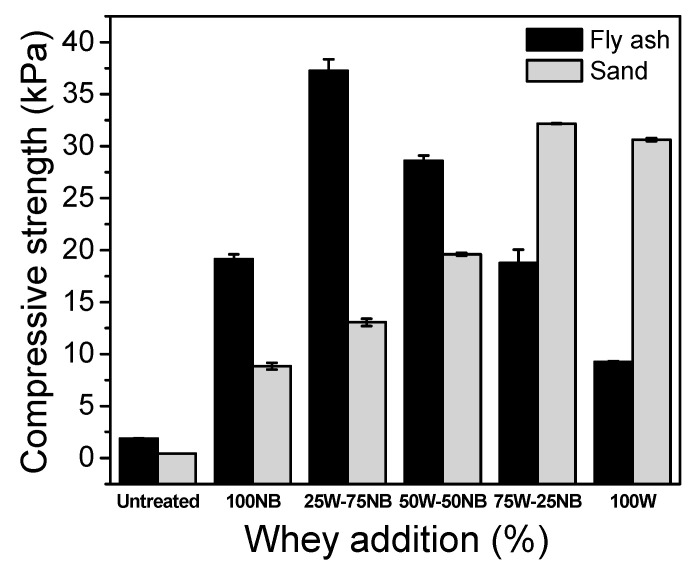
Compressive strength of sand and fly ash treated with different whey additions.

The bioconsolidation of sand and fly ash with different concentrations of whey is shown in Figure 6. The results revealed that the highest bioconsolidation degree was obtained with fly ash and 25 W-75 NB of 37.2 kPa. The increase in compressive strength could have been generated by biogenic CaCO_3_ deposition on the *S. pasteurii* surface and within filling material [53]. According to previous characterizations, XRD showed that biogenic CaCO_3_ produced by 25 W-75 NB assay had a high vaterite content (87.6%). This type of crystal has a low density and a large specific surface area, which might produce greater adsorption of filled material [54]. After 25% whey, a reduction in the CS trend was observed, probably caused by a vaterite content decrease. Whey has different kinds of components that seal the surface layer of fly ash, which hinders their transport through the column. For this reason, the entire fly ash column compressive strength is lower at high whey concentrations. Fly ash was very well consolidated with vaterite crystals of biogenic CaCO_3_ using 25 W-75 NB due to its fine granulometry (Table S2).

In sand bioconsolidation, compressive strength ranged between 8.8 and 32.2 kPa, and the best results were obtained with 75 W-25 NB and 100 W. Biogenic CaCO_3_ produced in 75 W-25 NB had a similar concentration of calcite (51.7%) and vaterite (48.3%), and with this mixture, the maximum compressive strength was obtained. Calcite crystals are rhombohedral, while vaterite crystals are spherical with the same size, which improves the filling of sand pores. A lowest specific surface area and pore volume of calcium carbonate favors a better CS in sand.

**Figure 7:**
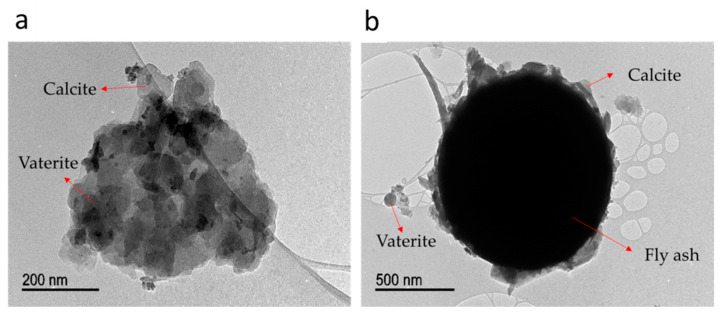
TEM micrographs of bioconsolidation of (**a**) sand, and (**b**) fly ash at 50 W-50 NB. These images show vaterite and calcite crystals on these material’s surface, such as biocement.

Figure 7a shows a TEM micrograph of sand bioconsolidation using 50% of whey. Although sand could not be observed (due to the large particle size), the biogenic calcium carbonate surrounding sand granule junctions was distinguished. Round crystals are the most common, but calcite is also observed. Some elongated black dots that correspond to microbial cells that act as crystal nucleation sites are also noted. Figure 7b shows a fly ash particle mainly surrounded by round crystals, although some straight sides of calcite can also be seen. With these results, it is proven that *S. pasteurii* isolated from agricultural soils could produce calcium carbonate in contact with calcium that is placed in the middle to carry out the bioconsolidation of sand and fly ash.

To reduce the environmental problem due to the release of large amounts of whey in rivers, two alternative uses were proposed. First, the use of whey as a microbial cultivation medium and, second, the potential application as a natural cement using fly ash. Conventionally, the biotechnology process involves the use of expensive media and sterile conditions, and this must be changed by inexpensive and soft production conditions in order to obtain accessible use of *Sporosarcina pasteurii*. It is necessary to find an economical substrate for the growth in bacteria with the production of high urease activity for large-scale production [19]. The cost of 1 L of media (nutrient broth) including urea was USD 2 in this work and there are other related costs such as water, energy and personnel. Sometimes, inexpensive media are generally subject to low quality control and reproducibility. However, it could be a good choice in order to diminish costs for large biotechnological production. Thus, whey can be an alternative growth media for *Sporosarcina pasteurii* isolated from soils.

It is well known in microbially induced calcium carbonate precipitation (MICP) that the crystal phase depends on different parameters such as bacteria strain, urease activity, calcium sources, and media [55,56,57]. In this work, we established that varying the concentration of whey on nutrient broth could modify the vaterite: calcite ratio by using, a higher amount of whey in the medium, which influences calcium carbonate purity (obtained by TGA results). This control of polymorphism produces a specific type of calcium carbonate phase which has relevant implications and applications. Vaterite has a high surface area as compared with calcite, which indicates that this polymorph could be employed as a catalyst/support in different reactions and for the absorption of heavy metals in wastewaters and contaminated soils. Likewise, these crystals could bring stability to minerals and they can be utilized in different industries, for example as a filler in thermoplastics or as composite materials, among others.

Consequently, the distinct CaCO_3_ morphologies affect the production of concrete building blocks by cementing natural fly ash. Our results demonstrate that sand and fly ash bioconsolidation could be employed in the construction industry. This depends on the particle size of the filler, as of the crystal morphology of carbonate obtained. With materials such as fly ash (small grain size), CaCO_3_ obtained with low whey concentrations in culture is optimum, while with high grain size materials such as sand, CaCO_3_ obtained from high whey quantities should be utilized. In all cases, both treatments increase compressive strength and their high thermal behavior (decomposition temperature above 600 °C) which would be advantageous in their potential use as a cement alternative. From an environmental point of view, this process could be an interesting alternative that will provide sustainability to construction processes. Bioconsolidation with *S. pasteurii* isolated from agricultural soils in Colombia could be an inexpensive alternative to treating these materials. However, before that, it is necessary to develop pilot-scale studies that verify the presented results in large-scale production, especially for the bioconsolidation of sand with 100 W. Future research directions could address the use of these polymorphs obtained by *Sporosarcina pasteurii* isolated from soils in different industries, such as for filler and catalysts.

## 4. Conclusions

Whey was successfully employed as an economical nutrient source for the growth of *Sporosarcina pasteurii* isolated from agricultural soils in Boyacá (Colombia). To the usual nutrient broth, various concentrations of whey were added. This generated different CaCO_3_ crystallizations. Thus, crystal morphology is influenced by whey incorporation because the production of calcite crystals is directly related to the presence of impurities present in the media. The increase in whey percentage in the culture media promoted calcite production with urea (30 g/L) and calcium chloride (30 g/L) at 200 rpm and 30 °C for 24 h. Bioconsolidation experiments evidenced that *S. pasteurii* increased compressive strength, probably due to the presence of biogenic CaCO_3_ on the material. This parameter depended on the different polymorphs formed as a kind of filler. Whey addition in nutrient broth promoted calcite production and this type of crystal is more stable and has a higher density compared to vaterite. This is important in sand bioconsolidation treatments. However, in fly ash columns, vaterite showed the best results because of its high specific surface area and lower density. From a materials perspective, this research opens an interesting double benefit perspective: using a wide surplus of whey to create solid structures (using costless natural ashes as filler material) instead of causing environmental problems.

## Figures and Tables

**Table 1:** Effect of whey concentration in nutrient broth on crystal size and specific surface area, pore size and pore volume of biogenic CaCO_3._

Treatment	Crystal Size Vaterite (nm) *	Crystal Size Calcite (nm) *	Specific Surface Area (m^2^/g)	Pore Volume (cm^3^/g)	Pore Size (nm)	Purity (%) **
100 NB	3.6	3.2	25	0.11	22.5	96
25 W-75 NB	3.6	3.2	23	0.04	8.6	91
50 W-50 NB	3.2	3.6	19	0.05	9.3	83
75 W-25 NB	3.2	3.2	6	0.01	24.8	81
100 W	3.8	2.7	2	0.01	24.8	80

* Crystallite size was determined by Scherrer equation using XRD. ** Purity determined by TGA results.

## Data Availability

Data is contained within the article or supplementary material.

## Supplementary Materials

The following are available online at https://www.mdpi.com/1996-1944/14/10/2470/s1, Figure S1: IR Spectra of CaCO_3_ produced with different whey treatments, Table S1: Whey composition used as culture media of *S. pasteurii* in comparison with other studies, Table S2: Composition of sand and fly ash used in bioconsolidation, Table S3: Information bands of IR spectra of biogenic CaCO_3_.
