# Correction: Chaparro et al. Whey as an Alternative Nutrient Medium for Growth of *Sporosarcina pasteurii* and Its Effect on CaCO_3_ Polymorphism and Fly Ash Bioconsolidation. *Materials* 2021, *14*, 2470

**DOI:** 10.3390/ma17112781

**Published:** 2024-06-06

**Authors:** Sandra Chaparro, Hugo A. Rojas, Gerardo Caicedo, Gustavo Romanelli, Antonio Pineda, Rafael Luque, José J. Martínez

**Affiliations:** 1 School of Chemical Sciences, Faculty of Sciences, Pedagogical and Technological University of Colombia, 150001 Tunja, Colombia; patricia.chaparro@uptc.edu.co (S.C.); hugo.rojas@uptc.edu.co (H.A.R.); gerardo.caicedo@uptc.edu.co (G.C.); 2 Research and Development Centre on Applied Sciences “Dr. Jorge Ronco” (CCT-La Plata-CONICET, CIC-PBA), National University of La Plata, 1900 La Plata, Argentina; gpr@quimica.unlp.edu.ar; 3 Departamento de Química Orgánica, Universidad de Córdoba, Ctra NNal IV-A, Km 396, E-14014 Córdoba, Spain; 4 Scientific Center for Molecular Design and Synthesis of Innovative Compounds for the Medical Industry, Peoples Friendship University of Russia (RUDN University), 117198 Moscow, Russia

The Editorial Office was made aware of an error in Figure S1 within the original publication [1]. In addition to the corrected figure, the authors have provided raw data that were presented to an Academic Editor for evaluation. The original figure will now be replaced. The authors state that the scientific conclusions are unaffected. This correction was approved by the Academic Editor. The original publication has also been updated.

## Figures and Tables

**Figure S1:**
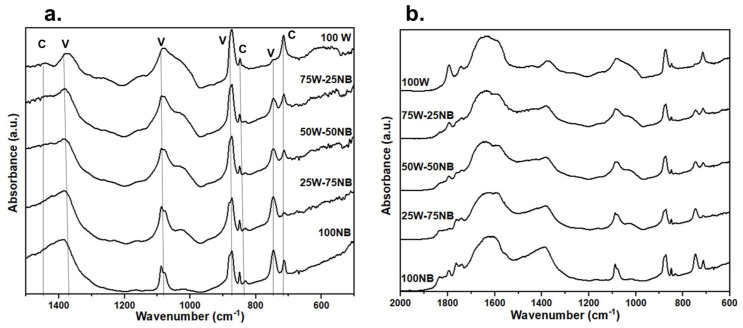
IR Spectra of CaCO_3_ produced with different whey treatments. (**a**) IR spectra fingerprint showing the characteristic bands of vaterite (V) and calcite (C) (**b**) IR spectra in the region of 2000–600 cm^−1^ where the presence of protein residues can be evidenced by the amide bands; C=O stretching mode of the amide functional group (1600–1700 cm^−1^), and N–H bending and C–N stretching vibrations (1500–1600 cm^−1^).

## References

[1] Chaparro S., Rojas H.A., Caicedo G., Romanelli G., Pineda A., Luque R., Martínez J.J. (2021). Whey as an Alternative Nutrient Medium for Growth of *Sporosarcina pasteurii* and Its Effect on CaCO_3_ Polymorphism and Fly Ash Bioconsolidation. Materials.

