# Supplementary material for: Whey as an Alternative Nutrient Medium for Growth of Sporosarcina pasteurii and Its Effect on CaCO3 Polymorphism and Fly Ash Bioconsolidation
Source: Materials (Basel). 2021 May 11;14(10):2470. doi: 10.3390/ma14102470 (PMC8151748; doi:10.3390/ma14102470)
Supplement: Supplementary file 1 [file materials-14-02470-s001.zip › materials-1194532-supplementary.pdf]

materials

MDPI

Supplementary materials

Whey as an Alternative Nutrient Medium for Growth of *Sporosarcina pasteurii* and Its Effect on $CaCO_3$ Polymorphism and Fly Ash Bioconsolidation

Sandra Chaparro [1], Hugo A. Rojas [1], Gerardo Caicedo [1], Gustavo Romanelli [2], Antonio Pineda [3,4], Rafael Luque [3,4,*] and José J. Martínez [1,*]

1 School of Chemical Sciences, Faculty of Sciences, Pedagogical and Technological University of Colombia, 150001 Tunja, Colombia; patricia.chaparro@uptc.edu.co (S.C.); hugo.rojas@uptc.edu.co (H.A.R.); gerardo.caicedo@uptc.edu.co (G.C.)

2 Research and Development Centre on Applied Sciences "Dr. Jorge Ronco" (CCT-La Plata-CONICET, CIC-PBA), National University of La Plata, 1900 La Plata, Argentina; gpr@quimica.unlp.edu.ar

3 Departamento de Química Orgánica, Universidad de Córdoba, Ctra NNal IV-A, Km 396, E-14014 Córdoba, Spain; q82pipia@uco.es

4 Scientific Center for Molecular Design and Synthesis of Innovative Compounds for the Medical Industry, Peoples Friendship University of Russia (RUDN University), 117198 Moscow, Russia

* Correspondence: rafael.luque@uco.es (R.L.); jose.martinez@uptc.edu.co (J.J.M.); Tel.: +34-957211050 (R.L.)

Table S1. Whey composition used as culture media of *S. pasteurii* in comparison with other studies.

Component	Actual Work	Achal et al. [1]	Gabriec et al. [2]
pH	6.3	6.2	-
Kcal	22.2	-	-
Moisture (%)	94.200 ± 0.011	94.5	9.8
Solids (%)	5.8 ± 0.050	5.5	90.2
Protein (%)	1.900 ± 0.012	8.0	10.8
Fat (%)	0.100 ± 0.020	2.0	0.3
Ash (%)	0.400 ± 0.015	0.5	8.8
Ca (mg/L)	0.580 ± 1.141	353	16.8

Table S2. Composition of sand and fly ash used in bioconsolidation.

Parameter	Value
**Sand**	
pH	8.80
Organic matter (%)	0.18
Phosphorus (ppm)	12.70
Iron (ppm)	5.39
Manganese (ppm)	0.07
Cupper (ppm)	1.17
Sodium (ppm)	0.20
Grain size	Retained in sieve of de 0.50 mm
Mineralogy	quartz
**Fly Ash**	
SiO_2	53.17
CaO	4.62
Al_2O_3	33.72
MgO	0.67
Fe_2O_3	3.16
K_2O	1.46

Na_2O	0.75
SO_3	0.61
TiO_2	1.22
Grain Size	Retained in sieve of 0.45 μm

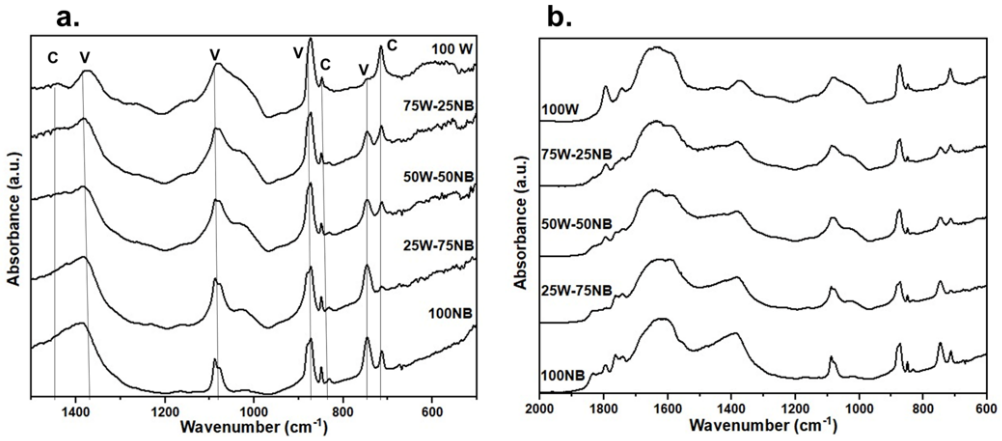

Figure S1. IR Spectra of $CaCO_3$ produced with different whey treatments. (a) IR spectra fingerprint showing the characteristic bands of vaterite (V) and calcite (C) (b) IR spectra in the region of 2000–600 cm^{-1} where the presence of protein residues can be evidenced by the amide bands; C=O stretching mode of the amide functional group (1600-1700 cm^{-1}), and N–H bending and C–N stretching vibrations (1500–1600 cm^{-1}).

Table S3. Information bands of IR spectra of biogenic $CaCO_3$.

Band	Assignation	Polymorph*
875 cm^{-1}	ϑ_2 = CO_3 out of plane deformation mode	C, V
713 cm^{-1}	ϑ_4 = OCO bending (in-plane deformation) mode	C
1440 cm^{-1}	ϑ_3 = asymmetric C-O stretching mode	C
745 cm^{-1}	ϑ_4 = OCO bending (in-plane deformation) mode	V
1440–1490 cm^{-1} divided peak	ϑ_3 = asymmetric C-O stretching mode	V
1077 cm^{-1}	ϑ_1 = symmetric C-O stretching mode	V
1600–1700 cm^{-1}	C=O stretching mode of the amide functional group	AM
1500–1600 cm^{-1}	N-H bending and C-N stretching vibrations	AM

* V = vaterite, C = calcite, AM = amide bands of protein residues Refs: [3–6], $CaCO_3$ normal vibrations are ϑ_1 = symmetric C-O stretching mode, ϑ_2 = CO_3 out of plane deformation mode, ϑ_3 = asymmetric C-O stretching mode and ϑ_4 = OCO bending (in-plane deformation) mode.

Reference

1. Achal, V., Mukherjee, A., Basu, P.C., Reddy, M.S. Lactose mother liquor as an alternative nutrient source for microbial concrete production by *Sporosarcina pasteurii*. *J Ind Microbiol Biotecno.* **2009,** *36*, 433–438.
2. Grabiec, A.M., Klama, J., Zawal, D., Krupa, D. Modification of recycled concrete aggregate by calcium carbonate biodeposition. *Constr. Build Mater.* **2012,** *34*, 145–150.
3. Al, Omari M.M., Rashid, I.S., Qinna, N.A., Jaber, A.M., Badwan, A.A. *Calcium Carbonate*. 1st ed.; Elsevier: Amsterdam, The Netherlands, **2016**; In Profiles of drug substances, excipients and related methodology, pp. 31–132.
4. Andersen, F.A., Brečević, L. Infrared spectra of amorphous and crystalline calcium carbonate. *Acta Chem. Scan.* **1991**, *45*, 1018–1024.
5. Seshadri, S., Khurana, R., Fink, A. Fourier Transform Infrared Spectroscopy in Analysis of Protein Deposits. *Methods Enzymol.* **1999**, *309*, 559–576.
6. Lei, Y., Zhou, Q., Zhang, Y., Chen, J., Sun, S., Noda, I. Analysis of crystallized lactose in milk powder by Fourier-transform infrared spectroscopy combined with two-dimensional correlation infrared spectroscopy. *J. Mol. Struct.* **2010**, *974*, 88–93.
